# Supplementary material for: The extracellular chaperone Clusterin enhances Tau aggregate seeding in a cellular model
Source: Nat Commun. 2021 Aug 11;12:4863. doi: 10.1038/s41467-021-25060-1 (PMC8357826; doi:10.1038/s41467-021-25060-1)
Supplement: Supplementary file 2 — Reporting Summary [file 41467_2021_25060_MOESM2_ESM.pdf]

nature research

Corresponding author(s): F. Ulrich Hartl

Last updated by author(s): Jul 1, 2021

Reporting Summary

Nature Research wishes to improve the reproducibility of the work that we publish. This form provides structure for consistency and transparency in reporting. For further information on Nature Research policies, see our Editorial Policies and the Editorial Policy Checklist.

Statistics

For all statistical analyses, confirm that the following items are present in the figure legend, table legend, main text, or Methods section.

n/a	Confirmed	
☐	☒	The exact sample size (*n*) for each experimental group/condition, given as a discrete number and unit of measurement
☐	☒	A statement on whether measurements were taken from distinct samples or whether the same sample was measured repeatedly
☐	☒	The statistical test(s) used AND whether they are one- or two-sided<br>*Only common tests should be described solely by name; describe more complex techniques in the Methods section.*
☒	☐	A description of all covariates tested
☐	☒	A description of any assumptions or corrections, such as tests of normality and adjustment for multiple comparisons
☐	☒	A full description of the statistical parameters including central tendency (e.g. means) or other basic estimates (e.g. regression coefficient) AND variation (e.g. standard deviation) or associated estimates of uncertainty (e.g. confidence intervals)
☐	☒	For null hypothesis testing, the test statistic (e.g. *F*, *t*, *r*) with confidence intervals, effect sizes, degrees of freedom and *P* value noted<br>*Give P values as exact values whenever suitable.*
☒	☐	For Bayesian analysis, information on the choice of priors and Markov chain Monte Carlo settings
☒	☐	For hierarchical and complex designs, identification of the appropriate level for tests and full reporting of outcomes
☒	☐	Estimates of effect sizes (e.g. Cohen's *d*, Pearson's *r*), indicating how they were calculated

Our web collection on statistics for biologists contains articles on many of the points above.

Software and code

Policy information about availability of computer code

Data collection	SymPhoTime 64 (PicoQuant)<br>Attune NxT Software v 2.4-3.1<br>FACSDiva Version 6.1.3<br>Leica LAS X 3.5.7.23225 (Leica microsystems)<br>MAPS 2.1 software (Thermo Fisher)<br>FluorEssence V3.9 (HORIBA)<br>SerialEM 3.7.0
Data analysis	SymPhoTime 64 (PicoQuant)<br>Sigma plot 14.0<br>GraphPrism6<br>Origin Pro 2019b<br>FlowJo V9 and V10.7.1 software (FlowJo LLC)<br>Image J 1.53a<br>Huygens Essentials 19.10 (Scientific Volume Imaging)<br>Volocity V6.3 (Quorum Technologies)<br>AIDA Image Analyzer v.4.27 (Elysia Raytest)

For manuscripts utilizing custom algorithms or software that are central to the research but not yet described in published literature, software must be made available to editors and reviewers. We strongly encourage code deposition in a community repository (e.g. GitHub). See the Nature Research guidelines for submitting code & software for further information.

Data

Policy information about availability of data

All manuscripts must include a data availability statement. This statement should provide the following information, where applicable:

- Accession codes, unique identifiers, or web links for publicly available datasets
- A list of figures that have associated raw data
- A description of any restrictions on data availability

All data supporting the findings of this study are available within the manuscript. Source data are provided with this paper.

Field-specific reporting

Please select the one below that is the best fit for your research. If you are not sure, read the appropriate sections before making your selection.

- [x] Life sciences
- [ ] Behavioural & social sciences
- [ ] Ecological, evolutionary & environmental sciences

For a reference copy of the document with all sections, see nature.com/documents/nr-reporting-summary-flat.pdf

Life sciences study design

All studies must disclose on these points even when the disclosure is negative.

Sample size	No sample size calculation was performed. At least three independent experiments were performed in all cases as per commonly accepted field standards and to enable statistical analysis.
Data exclusions	Data was excluded in case technical problems were detected during experiment performance.
Replication	A minimum of three independent replicates were conducted for all experiments. Specific number of independent replicates is stated in figure legends.
Randomization	Mouse embryos of both sexes were chosen randomly for neuronal cell cultures. For all other experiments randomization did not apply.
Blinding	No blinding was applied since data collection and analysis were not strongly dependent on subjective interpretation of the data. The findings are supported by quantitative measurements and statistical analysis when relevant.

Reporting for specific materials, systems and methods

We require information from authors about some types of materials, experimental systems and methods used in many studies. Here, indicate whether each material, system or method listed is relevant to your study. If you are not sure if a list item applies to your research, read the appropriate section before selecting a response.

Materials & experimental systems

n/a	Involved in the study	
[ ]	[x]	Antibodies
[ ]	[x]	Eukaryotic cell lines
[x]	[ ]	Palaeontology and archaeology
[ ]	[x]	Animals and other organisms
[x]	[ ]	Human research participants
[x]	[ ]	Clinical data
[x]	[ ]	Dual use research of concern

Methods

n/a	Involved in the study	
[x]	[ ]	ChIP-seq
[ ]	[x]	Flow cytometry
[x]	[ ]	MRI-based neuroimaging

Antibodies

Antibodies used	anti-phospho-Tau AT8 (MN1020, Thermo Fisher Scientific), anti-CHMP2A (10477-1-AP, Proteintech), anti-Galectin 8 (ab109519, Abcam), anti-MAP2 (NB300-213, Novus Biologicals), Tau/Repeat Domain antibody (2B11, TECAN), mouse monoclonal Clu-α antibody (sc-5289, Santa Cruz Biotechnology), anti-Tau-1 antibody (nclone PC1C6, MAB3420, MERCK), Tau monoclonal antibody (TAU-5, MA5-12808, Thermo Fisher Scientific), recombinant anti-Clusterin antibody (EPR2911, ab92548, abcam) and anti-alpha-Synuclein antibody (LB509, ab27766, abcam). Anti-Mouse IgG HRP (A4416, Merk), anti-rabbit IgG (whole molecule)–peroxidase antibody produced in goat (A9169, MERCK) Goat-anti Rabbit IgG Alexa Fluor 405 (A-31556, Thermo Fisher Scientific), F(ab')2-Goat anti-Mouse IgG Alexa Fluor 633 (A-21053, Thermo Fisher Scientific), Alexa Fluor 647 AffiniPure Donkey Anti-Chicken IgY (IgG) (H+L)(703-605-155, Jackson Immunoresearch).
Validation	Most antibodies are validated by commercial suppliers:

Immunoblotting:
- Anti-phospho-Tau AT8 (MN1020, Thermo Fisher Scientific): This Antibody was verified by Cell treatment to ensure that the antibody binds to the antigen stated. Referenced in 191 publications.
- Tau/Repeat Domain antibody (2B11, TECAN). This antibody was validated in-house by immunoblotting by comparing the Tau/Repeat Domain antibody signal with the GFP antibody signal (11814460001, Sigma) of HEK293T cell lysate from cells overexpressing TauRD-YFP and purified TauRD-YFP.
- Mouse monoclonal Clu-α antibody (sc-5289, Santa Cruz Biotechnology): Knockdown validation. Referenced in 38 articles.
- Anti-Tau-1 antibody (nclone PC1C6, MAB3420, MERCK): Specificity described in Szendrei GI et al, J Neurosci Res 1993 and Billingsley ML, Kincaid RL Biochem J 1997. Referenced in 94 publications.
- Tau monoclonal antibody (TAU-5, MA5-12808, Thermo Fisher Scientific): Referenced in 70 publications.
- Recombinant anti-Clusterin antibody (EPR2911, ab92548, abcam): Knockout validated. Referenced in 4 publications.
- Anti-alpha-Synuclein antibody (LB509, ab27766, abcam): Abpromise guarantee. Referenced in 51 publications.

Immunofluorescence:
- Anti-phospho-Tau AT8 (MN1020, Thermo Fisher Scientific): This Antibody was verified by Cell treatment to ensure that the antibody binds to the antigen stated. Referenced in 28 publications.
- Anti-CHMP2A (10477-1-AP, Proteintech): Knockdown/Knockout validated. Referenced in 12 publications.
- Anti-Galectin 8 (ab109519, Abcam): Abpromise guarantee. Referenced in 7 publications.
- Anti-MAP2 (NB300-213, Novus Biologicals): Knockdown validated. Referenced in 27 publications.

Eukaryotic cell lines

Policy information about cell lines

Cell line source(s)	SH-SY5Y cells were a gift from Konstanze Winklhofer and Joerg Tazelt (10.1038/emboj.2011.86) and originally purchased from DSMZ (ACC 209).<br>HEK293T were purchased from ATCC.
Authentication	No further authentication was performed.
Mycoplasma contamination	No contamination was found by light microscopy (DAPI staining).
Commonly misidentified lines (See ICLAC register)	None.

Animals and other organisms

Policy information about studies involving animals; ARRIVE guidelines recommended for reporting animal research

Laboratory animals	Primary cortical neurons were prepared from E15.5 CD-1 wild type mouse embryos of both sexes (breeding line MpiCrlIcr:CD-1). Mice were housed in a specific pathogen free facility at 22 ± 1,5 °C, 55 ± 5% humidity, 14-hour light / 10-hour dark cycle.
Wild animals	No wild animals were used in the study.
Field-collected samples	No field collected samples were used in the study.
Ethics oversight	All experiments involving mice were performed in accordance with the relevant guidelines and regulations of the Government of Upper Bavaria (Germany).

Note that full information on the approval of the study protocol must also be provided in the manuscript.

Flow Cytometry

Plots

Confirm that:

- [x] The axis labels state the marker and fluorochrome used (e.g. CD4-FITC).
- [x] The axis scales are clearly visible. Include numbers along axes only for bottom left plot of group (a 'group' is an analysis of identical markers).
- [x] All plots are contour plots with outliers or pseudocolor plots.
- [x] A numerical value for number of cells or percentage (with statistics) is provided.

Methodology

Sample preparation	Creation of stable cell lines expressing the corresponding α-Syn and Tau constructs fused to fluorescence proteins: SH-SY5Y and HEK293T cells were transfected using Lipofectamine (Thermo Fisher). Cells were cultured in in Dulbecco's modified Eagle's medium (DMEM, Biochrom) supplemented with 10 % fetal bovine serum (FBS, GIBCO), 2 mM L-glutamine (GIBCO) and 1,000 µg/ml geneticin for selection. Polyclonal and monoclonal cell lines were generated by fluorescence-activated cell sorting. Upon selection, cells were cultured in medium supplemented with 200 µg/ml geneticin (Thermo Fisher) and penicillin/streptomycin (Thermo Fisher).

	Quantification of FRET positive cells: Cells were harvested with TrypL Express Enzyme (Gibco), washed with PBS once and resuspended in PBS for analysis.
Instrument	Creation of stable cell lines: BD FACS Aria III (BD Biosciences) for cell sorting. FITC-A, DAPI-A lasers for YFP/GFP and mTurquoise2 detection, respectively.<br><br>Quantification of FRET positive cells: Attune NxT flow cytometer (Thermo Fisher Scientific) for FRET positive cells quantification. To measure mTurquoise2 and FRET fluorescence signals, cells were excited with 405 nm laser light and fluorescence was determined using 440/50 and 530/30 filters, respectively. To measure the YFP fluorescence signal, cells were excited at 488 nm and emission was recorded using a 530/30 filter.
Software	Creation of stable cell lines: FACSDiva Version 6.1.3.<br><br>Quantification of FRET positive cells: Analysis were performed with FlowJo V9 and V10.7.1.
Cell population abundance	Creation of stable cell lines: All sorted cells showed the corresponding fluorescence protein expression.<br><br>Quantification of FRET positive cells: For each sample, 50,000 single cells were collected and analyzed.
Gating strategy	Quantification of FRET positive cells: After gating single cells, an additional gate was introduced to exclude YFP-only cells that show a false-positive signal in the FRET channel due to excitation at 405 nm. The FRET positive gate was set by plotting the FRET fluorescence signal versus the mTurquoise2 fluorescence signal using as reference non-seeded cells.

☒ Tick this box to confirm that a figure exemplifying the gating strategy is provided in the Supplementary Information.
